# Natural Compounds and Healthy Foods: Useful Tools against Onset and Progression of Chronic Diseases

**DOI:** 10.3390/nu15132898

**Published:** 2023-06-27

**Authors:** Antonella Amato

**Affiliations:** Department of Biological-Chemical-Pharmaceutical Science and Technology (STEBICEF), University of Palermo, Viale delle Scienze, 90123 Palermo, Italy; antonella.amato@unipa.it; Tel.: +39-091-238975067

The Special Issue (SI) in Nutrients, titled “Natural Compounds and Healthy Foods: New Strategy to Counteract Chronic Diseases”, deals with the beneficial effects of some natural bioactive substances and the relative action mechanisms, providing evidence for the potential to counteract some chronic diseases (CD). The Special Issue includes three reviews and five original articles.

The prevalence of CD, such as obesity, diabetes, cardiovascular diseases, neurodegeneration, inflammatory intestinal diseases, and cancer, is currently increasing worldwide [1]. Pathogenesis involves complex interactions among genetic, metabolic, environmental factors, and pathogenic mechanisms, including oxidative stress [2] and inflammation [3]. Recent basic and clinical investigations have demonstrated the beneficial impact of different natural bioactive compounds present in foods or extracts derived from vegetables or microalgal biomass, in the prevention and treatment of obesity, diabetes, neurodegeneration, and cancer [4,5,6]. The bioactive compounds with a strong therapeutical potential belong to the class of natural antioxidants (such as tocopherols, polyphenols, and carotenoids), vegetal sterols (such as phytosterols), short and long-chain polyunsaturated fatty acids, bioactive peptides, and pre- and probiotics. A common mechanism of action underlying their beneficial effects is the ability to counteract cellular inflammation and oxidative damages, to prevent or ameliorate the consequent tissue dysfunctions. Accordingly, the SI review article from Cicio and colleagues [7] summarizes data from the literature regarding the beneficial potential of Brassicaceae extracts against intestinal bowel diseases (IBDs). The Brassicaceae are vegetables rich in flavonoids, phenylpropanoid derivatives, and glucosinolates, already known for their beneficial health effects against cancers [8]. The review summarizes the current knowledge about the potential anti-inflammatory activity of Brassicaceae-derived natural compounds and their possible effect in preventing and treating IBD. The review’s data collection reveals the ability of the Brassicaceae extracts to reduce the secretion of inflammatory and oxidant mediators by modulating the expression of pro-inflammatory transcription factor NFκB and to maintain the integrity of the intestinal barrier by positively modifying intestinal flora balance [7].

Further, an original article by Ricci and collaborators shows that geraniol, an acyclic monoterpene alcohol with well-known anti-inflammatory and antimicrobial properties, can mitigate irritable bowel syndrome (IBS) symptoms, by improving gut microbiota profiles. By performing a prospective, multicentric, randomized, double-blinded, placebo-controlled trial, the study evaluated symptom improvements and microbiota modulation in patients with IBS treated with a low-adsorbable geraniol food supplement (LAGS). The patients treated with geraniol showed a significant reduction in the IBS symptoms severity score (IBS-SSS) compared to the placebo with significant differences in the microbiota composition. Reduction in families, associated with an unhealthy profile, such as Oscillospira and Clostridiaceae and increase in taxa, positively associated with health conditions, such as Ruminococcaceae, were observed [9].

Among CD, obesity is a major public health concern because it increases the risk of developing diabetes, cardiovascular diseases, and neurodegeneration. Approaches for reducing the current obesity epidemic are becoming a primary focus of human health care. Therapeutic strategies for treating and preventing obesity and its related-metabolic dysfunctions include nutritional interventions focusing on consuming foods rich in anti-inflammatory compounds. The review article from Cao et al. systematically summarized the research progress of polyphenols in preventing obesity. In particular, the collected data focused on the molecular mechanisms by which dietary polyphenols target the mTOR signaling pathway. mTOR, a protein kinase that regulates cell growth, survival, metabolism, and immunity, seems to mediate the polyphenol’s anti-obesity effect by modulating lipid metabolism, adipogenesis, and inflammation [10]. 

To date, the most efficacious therapeutic interventions concern the prevention of obesity-related dysmetabolism, by applying a diet plan in which long-term nutraceutical consumption is included. This is demonstrated by the study of Terzo and colleagues [11] on human subjects, in which the researchers demonstrated that a *Cynara-cardunculus*-L.-based nutraceutical improved metabolic parameters in pre-obesity subjects. The subjects receiving the *Cynara cardunculus* supplementation showed significantly reduced body weight, glycemic, and lipid parameters and improved hepatic functionality, and cardiovascular parameters such as carotid-media thickness and endothelial function.

Another original article deals with the preventive action of the long-term intake of honey and D-limonene against the neurodegeneration occurring in High Fat Diet (HFD) obese mice. The results showed that honey and limonene ingestion, either separately or in combination, could counteract the HFD-dependent brain damage by reducing neuronal inflammation and oxidative stress, and by negatively modulating the expression of genes associated with AD [12]. 

Parkinson’s disease (PD) shares with AD some pathogenic mechanisms such as neuroinflammation, oxidative stress, and neuronal apoptosis. In the study of Lai and colleagues, the positive impact of p-Hydroxybenzyl alcohol (HBA), a phenolic compound extracted by *Gastrodiae Rhizoma*, against PD pathogenesis was examined. By using SHSY5Y cells treated with 6-hydroxydopamine (6-OHDA), a cellular model of PD, the researchers demonstrated that HBA reduced ROS overproduction, mitochondrial dysfunction, and cell death caused by 6-OHDA. The HBA neuroprotective effect may be associated with ROS-dependent JNK/Jun/caspase-3 signaling pathway inhibition [13]. 

Numerous in vitro and in vivo studies have confirmed that plant-derived alkaloids and flavonoids exhibit strong anticancer activity. Fisetin, a naturally occurring flavonoid present in various fruits and vegetables, is the topic of the review article by Kubina and collaborators. The review summarizes the chemopreventive and therapeutic effects, the molecular targets, and the mechanisms of action responsible for the fisetin-anticancer activity. The authors suggest that the flavonoid has a bioactive potential to become a complementary drug in the prevention and treatment of various cancerous conditions [14], including liver cancer. Regarding liver cancer, the most common risk factor for hepatocellular cancer development is chronic hepatitis B (CHB) infection, responsible for immune dysfunction and chronic hepatitis development. In the original study of Shiue and colleagues, the researchers evaluated if *Arthrospira* species, a cyanobacterium frequently used as a dietary supplement for its healthy nutritional profile, was able to reduce the hepatitis B surface antigen (HBsAg) in CHB patients under treatment with nucleoside analogs. Furthermore, oral *Arthrospira*-diet mice were also used to investigate the possible immunological mechanism of *Arthrospira* against the hepatitis B virus. The study results showed that *Arthrospira* treatment can reduce HBsAg and rejuvenate the immune tolerance in CHB patients, by inducing B, T, NK, and macrophage cell activation [15]. 

The studies collected in this Special Issue are useful in furthering the knowledge of the beneficial effects of healthy nutrition against the onset and progression of CD. Most results have also highlighted the biological mechanisms underlying the protective actions exerted by the extracts and natural compounds, providing a new basis for future investigations on new potential intervention strategies. These findings also help healthcare professionals to educate their patients and to facilitate their adoption of healthful eating behaviors.
